# Therapeutics for neurodegenerative diseases by targeting the gut microbiome: from bench to bedside

**DOI:** 10.1186/s40035-024-00404-1

**Published:** 2024-02-27

**Authors:** Yuan-Yuan Ma, Xin Li, Jin-Tai Yu, Yan-Jiang Wang

**Affiliations:** 1 Department of Neurology and Centre for Clinical Neuroscience, Daping Hospital, Third Military Medical University, Chongqing, 400042 China; 2 Institute of Brain and Intelligence, Third Military Medical University, Chongqing, 400042 China; 3 Chongqing Key Laboratory of Ageing and Brain Diseases, Chongqing, 400042 China; 4 Army 953 Hospital, Shigatse Branch of Xinqiao Hospital, Third Military Medical University, Shigatse, 857000 China; 5 Department of Neurology and National Center for Neurological Disorders, Huashan Hospital, State Key Laboratory of Medical Neurobiology and MOE Frontiers Center for Brain Science, Shanghai Medical College, Fudan University, Shanghai, 200040 China

**Keywords:** Neurodegenerative disease, Gut microbiome, Immune system, Vagus nerve, Circulatory system, Microbiome-targeted therapies

## Abstract

The aetiologies and origins of neurodegenerative diseases, such as Alzheimer’s disease (AD), Parkinson’s disease (PD), amyotrophic lateral sclerosis (ALS) and Huntington’s disease (HD), are complex and multifaceted. A growing body of evidence suggests that the gut microbiome plays crucial roles in the development and progression of neurodegenerative diseases. Clinicians have come to realize that therapeutics targeting the gut microbiome have the potential to halt the progression of neurodegenerative diseases. This narrative review examines the alterations in the gut microbiome in AD, PD, ALS and HD, highlighting the close relationship between the gut microbiome and the brain in neurodegenerative diseases. Processes that mediate the gut microbiome–brain communication in neurodegenerative diseases, including the immunological, vagus nerve and circulatory pathways, are evaluated. Furthermore, we summarize potential therapeutics for neurodegenerative diseases that modify the gut microbiome and its metabolites, including diets, probiotics and prebiotics, microbial metabolites, antibacterials and faecal microbiome transplantation. Finally, current challenges and future directions are discussed.

## Introduction

Neurodegenerative diseases (NDs) are characterized by the progressive loss of specific neuronal populations, resulting in motor and cognitive impairments [1]. Among these, Alzheimer’s disease (AD), Parkinson’s disease (PD), amyotrophic lateral sclerosis (ALS) and Huntington’s disease (HD) are common types of ND. The development of various NDs is influenced by ageing, genetic susceptibilities and environmental factors [2]. In addition, emerging evidence reveals that peripheral factors play vital roles in the development of NDs [3, 4]. The multifaceted aetiologies and origins of NDs suggest that it is insufficient to focus on the end pathology within the central nervous system to prevent the onset and progression of NDs.

**Fig. 1:**
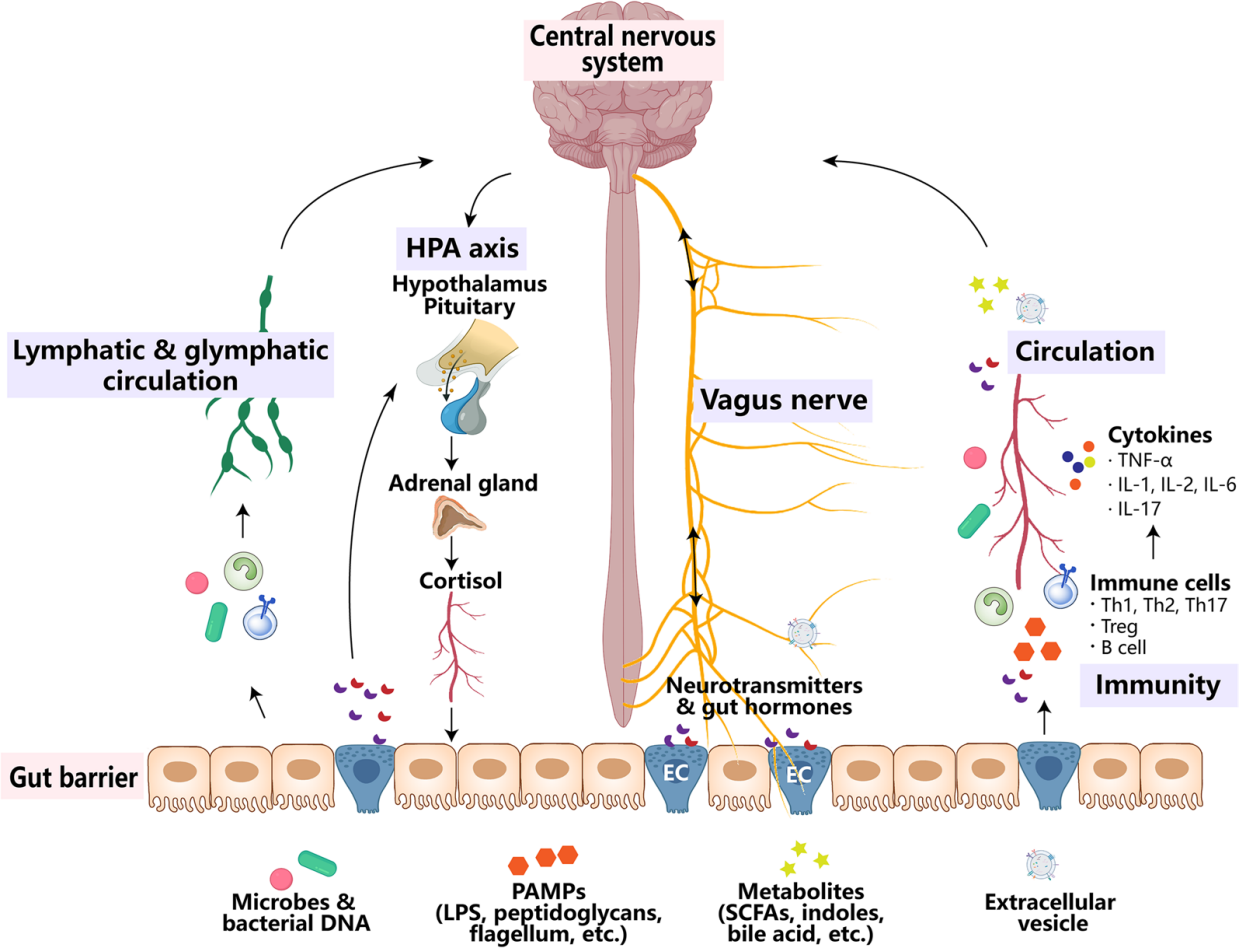
Overview of the gut microbiome-brain axis. Communications between the gut microbiome and the central nervous system (CNS) involve circulatory, immunological, vagus nerve, lymphatic and glymphatic, and neuro-endocrine (hypothalamic-pituitary-adrenal [HPA] axis) pathways. Gut microbes, their products (pathogen-associated molecular pattern [PAMPs] and extracellular vesicles) and metabolites, and gut neurotransmitters and hormones secreted by enteroendocrine cells, can transport to the CNS via peripheral circulation and impaired blood-brain barrier; they also interact with the host immune system and affect the CNS via blood circulation or lymphatic and glymphatic system. In addition, microbes and bacterial DNA can also translocate into the mesenteric lymphatic nodes. The vagus nerve transmits neural signals from the CNS to the gut, and also transports gut signals (activated by metabolites or neurotransmitters and hormones secreted by enteroendocrine cells) or gut microbiome-derived products like extracellular vesicles to the CNS. The HPA axis is a major neuro-endocrine system responding to stress by the release of cortisol from the adrenal cortex, which subsequently regulates the gut immune system and influences the gut microbiome composition. In turn, the gut microbiome can activate the HPA axis. *EC* enteroendocrine cell, *IL* interleukin, *LPS* lipopolysaccharide, *SCFAs* short-chain fatty acids, *Th* T helper cell, *TNF-α* tumour necrosis factor-α

The human gastrointestinal tract hosts trillions of microorganisms collectively known as the gut microbiome. Recent substantial evidence underscores the pivotal role of the gut microbiome in maintaining general health, especially health in the central nervous system [5, 6]. The gut microbiome is involved in the regulation of mental health, social behaviour, cognition function, etc., earning it the nickname “the second brain” of humans [5–7]. The composition of gut microbiome undergoes changes throughout the lifespan and is constantly influenced by factors such as ageing, genetic variants and environmental factors [8]. Notably, the gut microbiome has the capacity to regulate brain functions through the immunological pathway, the vagus nerve, the circulatory pathway, the hypothalamic-pituitary-adrenal axis, and the lymphatic and glymphatic circulation [9, 10] (Fig. 1). Many researchers believe that the crosstalk between the gut microbiome and the brain is crucial in the development of NDs, suggesting that modifying the gut microbiome holds promise as a therapeutic avenue for NDs [11–13].

The gut microbiome–brain axis in NDs has been discussed by several researchers in the past years [14–16]. In this review, we aim to summarize updated evidence implicating the critical role of the gut microbiome in the development of NDs and to highlight novel therapeutic opportunities that target the gut microbiome.

## Roles of the gut microbiome in NDs

Numerous pieces of evidence have shed light on the interactions between the gut and the brain in NDs. It has been reported that AD patients have a higher incidence of significant upper and lower gastrointestinal events, as nearly 80% of PD and 60% of ALS patients suffer from constipation [17–19]. HD patients are usually complicated with weight loss, gastritis, extended colonic transit times and nutritional deficiencies [20]. The gut microbiome has been proven to be a critical modulator of this gut–brain axis. Here, we will provide a brief overview of the gut microbiome–brain axis in NDs.

### Correlations between gut microbiome and NDs

In the past decade, clinical and preclinical studies have reported marked alterations in the gut microbiome in AD, PD, ALS and HD patients compared to their healthy controls [21, 22].

#### AD

A meta-analysis covering 378 healthy controls and 427 AD patients from 11 studies reported increased abundance of *Proteobacteria*, *Bifidobacterium* and *Phascolarctobacterium* and decreased abundance of *Firmicutes*, *Clostridiaceae*, *Lachnospiraceae* and *Rikenellaceae* in AD patients [23]. Recent studies have shown that the gut microbiome profiles are distinct between preclinical AD patients and healthy individuals, and the gut microbiome profiles and specific taxa are correlated with amyloid-β (Aβ) and tau pathological biomarkers in the brain and the plasma Aβ_42_/Aβ_40_ ratio, suggesting that changes of the gut microbiome may occur early in the disease process before neuronal injury [24, 25]. Furthermore, injection of Aβ into the lateral ventricle can cause cognitive impairments and inhibit the vagus nerve-mediated cholinergic anti-inflammatory pathway. This inhibition is accomplished by reducing the expression of M1-type acetylcholine receptors in the mouse brain [26]. Consequently, it results in decreased acetylcholine secretion and reduced α7 acetylcholine receptor levels in gut macrophages [26]. These changes shift the macrophages towards pro-inflammatory phenotypes, ultimately causing damage to enteric neurons and the gut mucosal barrier, and accelerating amyloidogenic pathways and gut microbiome dysbiosis [26]. These data suggest that Aβ in the brain has a direct effect on gut homeostasis and gut microbiome profiles.

The altered gut microbiome will further exacerbate the progression of AD. Transplantation of the gut microbiome derived from AD patients or mice (5×FAD) can worsen Aβ deposition, neuroinflammation and cognitive impairments in recipient mice compared to transplantation of the gut microbiome from healthy donors [11, 27, 28]. In addition, the deposition of Aβ, tau aggregates and cognitive impairments can be ameliorated by eliminating the gut microbiome in AD (APP/PS1, Tau/APOE4) mice through germ-free conditions or administration of antibiotics [29–31]. These studies have revealed that the gut microbiome and its products and metabolites play a significant role in altering the phenotypes and functions of microglia, thereby affecting Aβ deposition in the brains of AD patients.

#### PD

Increased abundance of *Akkermansia*, *Bifidobacterium* and *Lactobacillus* as well as reduced abundance of *Roseburia*, *Faecalibacterium*, *Blautia* and *Lachnospiraceae* have been mostly reported in PD patients and are strongly associated with the duration, onset time, and motor and nonmotor deficits of PD [32, 33]. Recently, Huang et al. investigated the gut microbiome across early PD, REM sleep behaviour disorder (RBD, prodromal PD), first-degree relatives of RBD (RBD-FDR, even earlier prodromal stage and younger population), and healthy controls. They found that the gut microbiome composition in RBD patients was altered to be close to early PD, with depletion of butyrate-producing bacteria and enrichment of *Collinsella*, *Desulfovibrio*, and *Oscillospiraceae UCG-005*. The RBD-FDR patients also showed the RBD/PD-like microbial alterations, including increased abundance of *Collinsella* and depletion of butyrate-producing bacteria. This evidence suggests that gut microbiome dysbiosis occurs at the early stage of PD and can be used to predict the onset of RBD and PD [34].

On the other hand, the gut microbiome can be a modifier in the progression of PD. Germ-free PD (α-synuclein-overexpressing) mice show improved motor and gastrointestinal functions and reduced insoluble α-synuclein aggregates in the brain compared to specific pathogen-free (SPF) PD mice. In addition, postnatal colonization of the germ-free PD mice recapitulated the genotype effects observed in SPF mice [35]. Furthermore, aggravation of microglial activation caused by gut microbiome-derived short-chain fatty acids (SCFAs) may lead to increased α-synuclein aggregates in PD mice [35].

#### ALS

Different cohorts of ALS patients have shown increased abundance of *Bacteroidetes* and *Enterobacteriaceae* and decreased abundance of *Lachnospiraceae* [36]. In addition, a lower ratio of *Firmicutes*/*Bacteroidetes* was found in ALS patients with cognitive impairments in comparison to those with normal cognition [37]. Administration of low-dose antibiotics exacerbates motor deficits and reduces survival in the ALS (SOD1^G93A^) mouse model. This effect may be attributed to the downregulation of homeostatic genes and upregulation of ND genes in spinal cord microglia in the ALS mice [38].

#### HD

Wasser et al. investigated the gut microbiome in HD gene expansion carriers (HDGECs) and their healthy controls, and found lower abundance of *Firmicutes*, *Lachnospiraceae* and *Akkermansiaceae* in HDGEC males [22]. Du et al. revealed that the HD patients have increased α-diversity and β-diversity of gut microbiota compared to healthy controls. In addition, the abundance of genus *Intestinimonas* is positively correlated with plasma levels of interleukin (IL)-4, and the abundance of genus *Bilophila* is negatively correlated with proinflammatory IL-6 levels, indicating that alterations in faecal microbiota may interact with the immune responses in HD patients [39]. In addition, it has been reported that faecal microbiome transplantation (FMT) from healthy (wild-type) into HD (R6/1) mice with antibiotics pretreatment significantly improves the cognitive functions only in females, which might be due to the microbial instability, acetate imbalance and gut immune profiles in male HD mice [40].

In conclusion, the gut microbiome–brain axis is bidirectional and complex, making it difficult to identify cause and effect between gut dysbiosis and neurodegeneration. The alterations in the gut microbiome in NDs are not always consistent across studies, possibly due in part to geographical, racial, or lifestyle differences. However, a common alteration seems to be the loss of butyrate-producing bacteria, such as *Lachnospiraceae* in AD, PD, ALS and HD, and *Roseburia* and *Faecalibacterium* in PD. The abundance of *Lachnospiraceae* is involved in ageing and the regulation of inflammation. It has been found that Rehmannia glutinosa polysaccharides can enhance the antioxidant enzyme system and delay ageing in *Caenorhabditis elegans*, which may be associated with increased abundance of *Lachnospiraceae NK4B4* group and regulation of amino acid metabolism and energy cycling [41]. FMT from young mice rejuvenates the aged hematopoietic stem cells and mitigates inflammatory signals by increasing *Lachnospiraceae* and tryptophan-associated metabolites [42]. *Lachnospiraceae* also contributes to intestinal protection through enhancement of the L-lysine fermentation pathway in a mouse model of sepsis [43]. In addition, oral FMT leads to enrichment of *Lachnospiraceae* and butyrate, and suppresses liver ferroptosis via activation of the adenosine 5’-monophosphate-activated protein kinase/P62/nuclear factor erythroid 2-related factor 2 signalling and induction of mitophagy, subsequently mitigating the acetaminophen-induced liver injury in mice [44].

Oxidative stress is considered a part of the ageing process and is also involved in the development and progression of NDs [10]. A cross-sectional study has revealed that patients with NDs display elevated serum levels of soluble NADPH oxidase 2-dp, H_2_O_2_ and lipopolysaccharide (LPS) (which is derived from gram-negative bacteria). Furthermore, there is a positive correlation between the serum LPS and serum zonulin (a maker of gut permeability) levels, suggesting the involvement of gut barrier impairment and bacteria translocation in the elevation of oxidative stress in NDs [45]. It has been found that both commensal and pathogenic bacteria in gut can alter the oxidative stress by regulating mitochondrial activity [46]. *Lactobacillus* and *Bifidobacterium* are capable of converting nitrate and nitrites into nitric oxide [46]. Considering that the abundance of *Lactobacillus* and *Bifidobacterium* is increased in AD and PD patients, there may be higher nitric oxide concentrations in these patients, resulting in detrimental effects by the production of reactive oxygen species and reactive nitrogen species (such as superoxide and H_2_O_2_), thereby aggravating the disease. These pieces of evidence indicate that the gut microbiome plays a role in the oxidative stress in NDs, and may represent a promising therapeutic target for NDs.

## Pathways by which the gut microbiome communicates with the brain in NDs

**Fig. 2:**
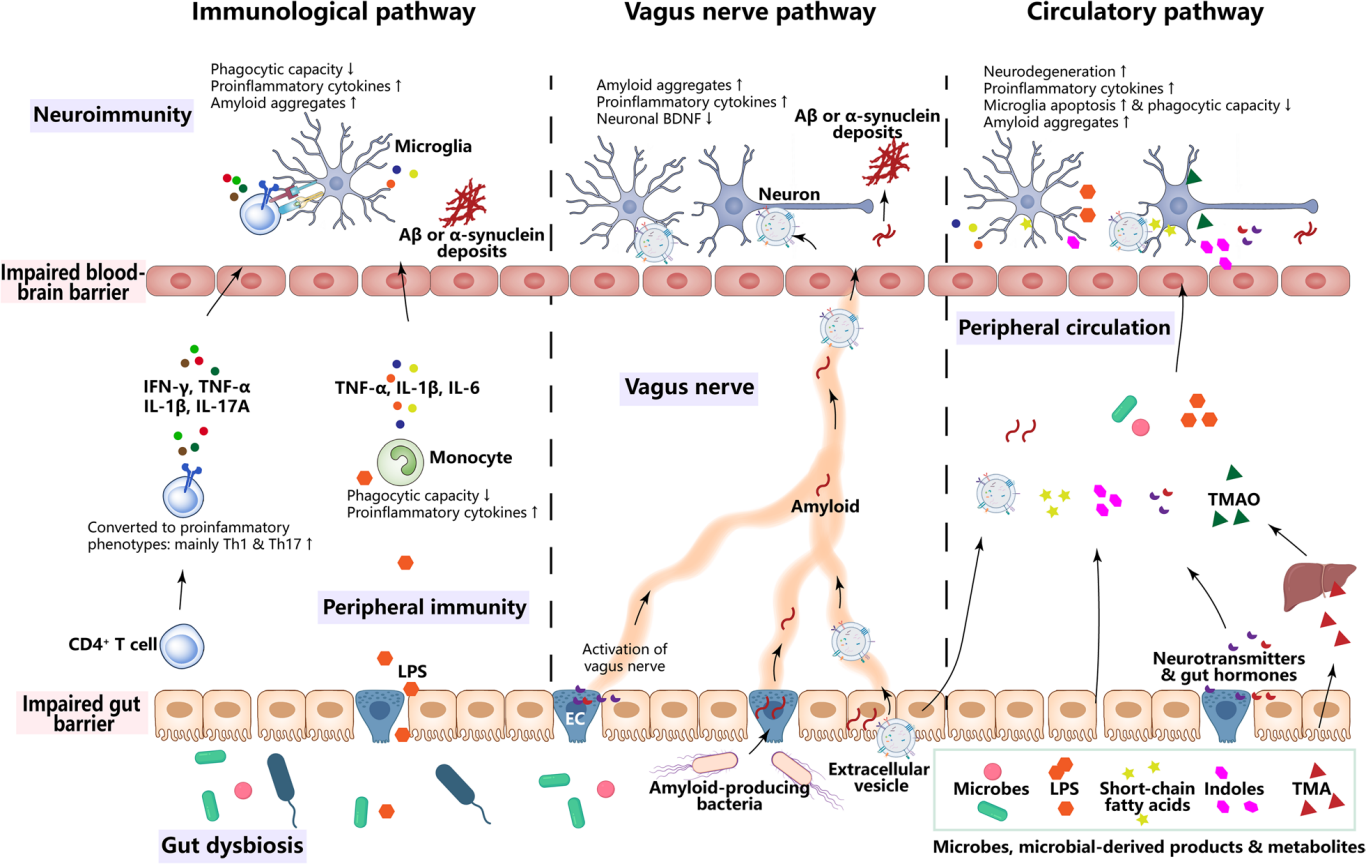
Routes by which microbes modulate neurodegenerative diseases. In the immunological pathway, gut microbe-derived products or metabolites (e.g., LPS) pass through the gut barrier and activate innate immune cells, primarily monocytes, and subsequently increase secretion of proinflammatory cytokines (TNF-α, IL-1β, IL-6, etc.) and reduce the phagocytic capacity of monocytes for amyloid proteins, leading to elevated neuroinflammation and amyloid aggregates in the brain. Gut dysbiosis also promotes the conversion of CD4^+^ T cells into proinflammatory phenotypes (Th1 and Th17), which secrete proinflammatory cytokines (IFN-γ, TNF-α, IL-1β, IL-17 A, etc.). These microbes can traverse the impaired blood-brain barrier and interact with microglia in the brain, exacerbating neuroinflammation. In the vagus nerve pathway, neurotransmitters and hormones secreted by enteroendocrine cells upon stimulation by the gut microbiome and its products can activate the vagus nerve. Amyloid-producing bacteria trigger gut amyloid accumulation, which is retrogradely transported to the brain via the vagus nerve. Gut microbe-derived extracellular vesicles can penetrate the brain through the vagus nerve and bloodstream, reducing BNDF expression in neurons and exacerbating neuroinflammation. In the circulatory pathway, microbes, their products, and neurotransmitters and hormones secreted by enteroendocrine cells can enter the peripheral circulation and cross the blood-brain barrier, leading to damage to neurons and microglia, reduced microglial phagocytic capacity, and increased neuroinflammation and amyloid aggregates in the brain. *BDNF* brain-derived neurotrophic factor, *EC* enteroendocrine cell, *IFN-γ* interferon-γ, *IL* interleukin, *LPS* lipopolysaccharide, *Th* T helper cell, *TMA* trimethylamine, *TMAO* trimethylamine-N‐oxide, *TNF-α* tumour necrosis factor-α

The complex interplay between the gut microbiome and the brain in NDs may occur through immunological, vagus nerve and circulatory pathways (Fig. 2).

### Immunological pathway

The brain and the periphery, as well as the innate and adaptive immunity, are important modulators of neuronal health in NDs [47–49]. Senescence of immune cells and chronic inflammation are indicators of brain ageing and NDs [50]. Due to the increased oxidative stress and mitochondrial dysfunction, the microglia in brain or the peripheral monocytes become less efficient in the removal of disease-specific protein aggregates, such as Aβ in AD, α-synuclein in PD and huntingtin protein in HD, and induce excessive inflammation [3, 48, 50, 51]. Importantly, the gut microbiome plays a role in the development and function of microglia and monocytes [52, 53]. It was found that germ-free mice display altered cell proportions and immature phenotypes of microglia, indicating that gut microbiome is involved in the normal development of microglia [53, 54]. The microglia in aged SPF mice display altered phenotypes and increased levels of oxidative stress compared to those in young SPF mice, while there is no such difference between young and aged germ-free mice. This result suggests that the gut microbiome regulates the ageing of microglia [55]. In addition to microglia, gut dysbiosis also influences the lifespan of monocytes, evidenced by the increased number of apoptotic Ly6C^high^ monocytes in the peripheral [56, 57]. Additionally, the products of gut microbiome (LPS, flagellin, etc.) in the blood can regulate the functions of trafficking monocytes [58]. In NDs, transplantation of the faecal microbiome from healthy donors has been shown to ameliorate microglial reactivity and functional impairments in both AD and PD mice, and decrease the levels of circulating blood inflammatory monocytes in AD mice [59].

Currently, many studies have revealed the role of peripheral T cells in the pathogenesis of NDs, although these diseases are not predominantly characterized by immune cell infiltration into the central nervous system as seen in conditions like multiple sclerosis or infections. T cells infiltrating into the brain may interact with microglia, aggravating neuroinflammation and contributing to the pathogenesis of NDs, such as AD and PD [60–63]. A recent study highlighted the essential role of peripheral activation of conventional CD4^+^ T cells by the gut microbiome in microglial maturation, proper synaptic pruning, and cognitive function in mice [64]. Oral pathogens *Veillonella parvula* and *Streptococcus mutans* can aggravate the motor deficits and neurodegeneration in MPTP-induced PD mice, which may be due to the alterations of gut microbiome, increased microglial activation and Th1 cell infiltration into the brain [65].

### Vagus nerve pathway

The vagus nerve connects the gut’s muscular and mucosal layers with the brainstem. Notably, the vagus nerve is crucial for transmitting signals from the gut microbiome to the brain. An animal study found that FMT from aged mice or human donors into young mice impaired the hippocampus-dependent memory, while increasing the vagal ascending activity ameliorated this adverse effect [66]. This finding suggests that vagus nerve stimulation holds potential as a treatment for alleviating cognitive impairments associated with gut dysbiosis. Consistently, human data have revealed that vagus nerve stimulation is a possible therapy for AD and PD patients [67, 68]. Stimulation of the vagus nerve can improve the cognitive functions and decrease the Aβ loads in the hippocampus of 6-month-old APP/PS1 transgenic mice by promoting microglial phagocytic activity towards Aβ [69]. Furthermore, it has been reported that neurotransmitters and gut hormones secreted by enteroendocrine cells induced by the gut microbiome can influence neuroinflammation and cognitive function through vagus nerve stimulation [70, 71].

In AD and PD, Aβ and α-synuclein are deposited not only in the brain but also in the gut [72–74]. A variety of amyloid-producing bacteria in the gut can trigger amyloid accumulation, and these nonhuman amyloids with prion-like properties can undergo retrograde translocation to the brain via the enteric nerve and the vagus nerve [75–77]. Additionally, gut microbiome-derived extracellular vesicles can penetrate both intestinal epithelium and vascular endothelium, and enter host cells through endocytosis [78]. It has been found that the gut microbe-derived extracellular vesicles can enter the brain via blood and the vagus nerve, subsequently affecting cognitive function in mice [79]. These findings shed light on why full truncal vagotomy is associated with a reduced risk of PD [80, 81].

The protective effects of vagus nerve stimulation and vagotomy on NDs appear to be a contradiction. We hypothesize that this could be explained that the vagus nerve serves as a conduit for not only beneficial signals but also potentially harmful substances. It is speculated that vagus nerve stimulation, in the condition of eradication of gut pathogens, might be an effective therapeutic approach for NDs.

### Circulatory pathway

Microbe-derived products and metabolites play important roles in the gut microbiome–brain communication. In fact, the blood is not “sterile” even in healthy organisms, and it is speculated that gut is a major source of the microbes in blood [82]. Interestingly, it has been found that the microbiome profiles in blood are distinct between mild cognitive impairment or AD patients and healthy controls [83]. LPS, a natural ligand for Toll-like receptor 4 (TLR4), is a major surface membrane component of gram-negative bacteria. LPS can translocate into the blood and the brain through the impaired gut barrier and blood-brain barrier (BBB), subsequently activating TLR4 on microglia and exacerbating amyloid aggregation and progression of NDs [84–86]. Consistently, colocalization of LPS and Aβ plaques has been observed in the postmortem brain tissues of AD patients [87]. Moreover, many microbial metabolites can enter systemic circulation and directly influence brain functions [71]. For example, acetate, a SCFA, is increased in the blood of AD patients, and can reduce microglial phagocytosis of Aβ and accelerate disease progression in AD mice [85, 88]. High levels of the gut microbial metabolite trimethylamine-N‐oxide (TMAO) in the blood have been found to exacerbate brain pathology associated with AD and PD and aggravate neuroinflammation in mouse models [89, 90].

Altogether, the above evidence indicates that the gut bacteria and their products and metabolites communicate with the brain via immunological, vagus nerve and circulatory system pathways. Further studies are needed to detail the mechanisms involved in these processes. The gastrointestinal tract harbours a complex ecosystem, including microorganisms and their products and metabolites. We propose that the gut microbiome impacts NDs through these routes simultaneously, and modulation of the gut microbiome is a promising approach to targeting multiple key pathways involved in the pathogenesis of NDs.

## Potential clinical approaches targeting the microbiome in NDs

**Fig. 3:**
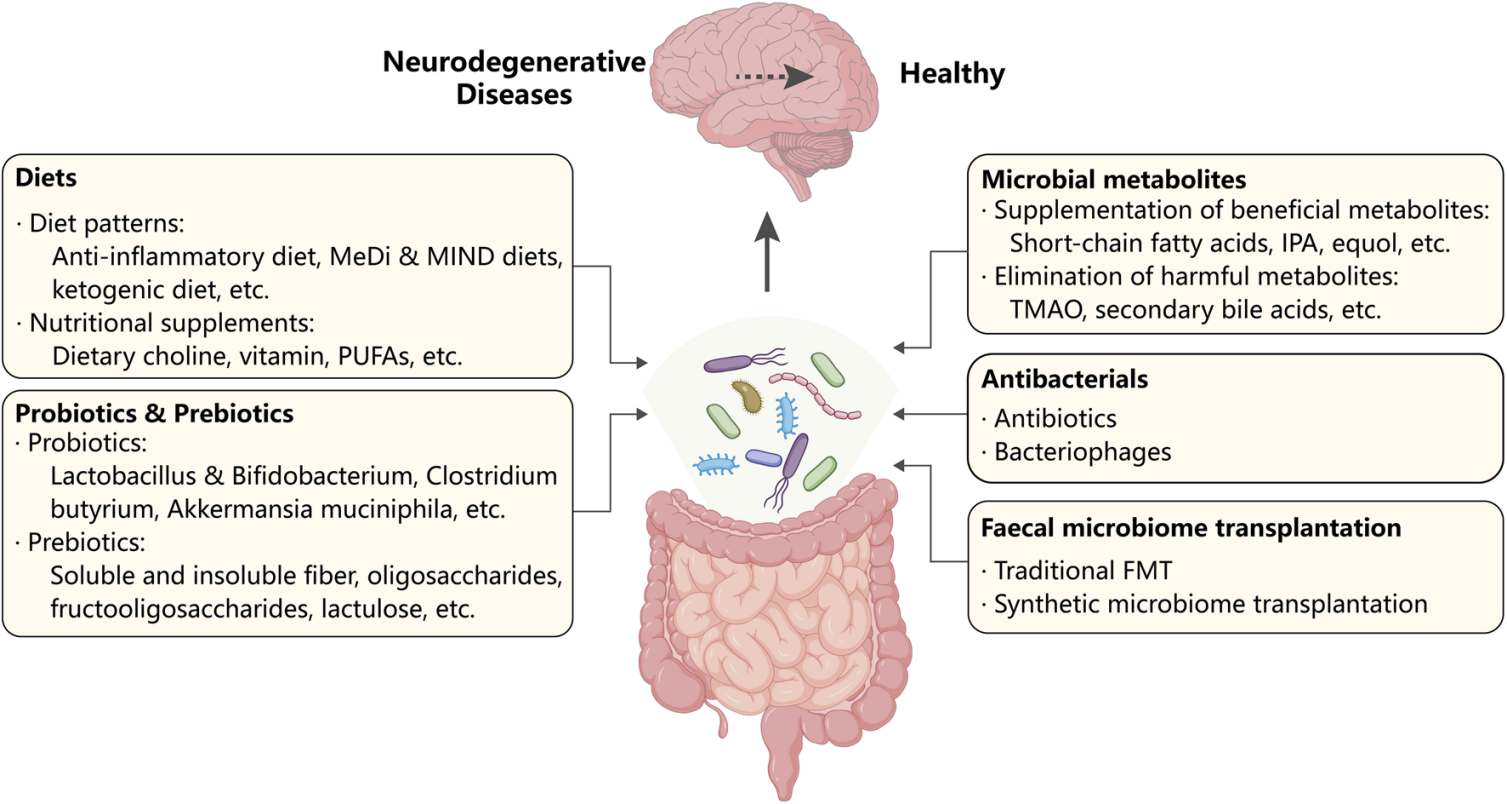
Potential strategies of gut microbiome-targeting therapies for neurodegenerative diseases. Diet (diet patterns and nutritional supplements), probiotics and prebiotics, microbial metabolites (supplementation of beneficial metabolites and elimination of harmful metabolites), antibacterials (antibiotics and bacteriophages) and faecal microbiome transplantation (traditional and synthetic microbiome transplantation) can all make the gut microbiome “healthy”, which prevents and alleviates neurodegenerative diseases. *FMT* faecal microbiome transplantation, *IPA* indole-3-propionic acid, *MeDi* Mediterranean, *MIND* Mediterranean-DASH Diet Intervention for Neurodegenerative Delay, *PUFA* polyunsaturated fatty acid, *TMAO* trimethylamine-N-oxide

The gut microbiome is a crucial modifier of ND risk, and targeting the gut microbiome can be a feasible approach to treating NDs. There are several factors and treatments that can reshape the gut microbiome, serving as potential therapies for NDs (Fig. 3).

### Diets

Diet is a significant determinant for the composition and function of the gut microbiome. It provides “fuel” for both hosts and microbes, and diet is also a crucial modifiable risk factor for NDs [91, 92]. This interplay between diet and microbiome may partly explain why diet can influence the development of NDs.

#### Anti-inflammatory diets

Recent studies have found that proinflammatory diets reflected by higher dietary Inflammatory Index scores are associated with higher incidences of AD and PD [93–95]. Importantly, the overall inflammatory potential of the diet is related to the gut microbiome composition. Certain bacteria including *Ruminococcus torques*, *Eubacterium nodatum*, *Acidaminococcus intestini* and *Clostridium leptum*, are more abundant in pro-inflammatory diets, while *Akkermansia muciniphila* is enriched in anti-inflammatory diets [96].

#### Mediterranean (MeDi) diet and Mediterranean-DASH Diet Intervention for Neurodegenerative Delay (MIND) diet

MeDi diet and MIND diet emphasize the consumption of plant foods, seafood, poultry, olive oil and dairy products while limiting the intake of red meat and high-saturated-fat foods. Several studies have shown that these diets can lower the risks of AD and PD and alleviate disease symptoms [97–101]. A systematic review revealed that the MeDi diet can increase the relative abundance of certain bacteria, such as *Christensenellaceae*, *Bilophila*, and *Escherichia/Shigella*, in both AD and PD patients, although the pathologies of the two diseases are different [102].

#### Ketogenic diet

The ketogenic diet is a high-fat, low-carbohydrate and adequate-protein diet. It has been applied in various neurological diseases, including epilepsy, AD and PD. Randomized trials have suggested that the ketogenic diet can improve cognitive function and quality of life in AD patients, as well as the motor and nonmotor symptoms in PD [103, 104]. Evidence from seizure studies highlights the vital role of the gut microbiome in the neuroprotective effects of a ketogenic diet. It has been reported that the germ-free mice or mice treated with antibiotics are resistant to the ketogenic diet-mediated seizure protection, and transplantation of the ketogenic diet gut microbiome can confer seizure protection to mice via reducing systemic gamma-glutamylated amino acids and increasing hippocampal γ-aminobutyric acid (GABA)/glutamate levels [105].

#### Nutritional supplements

##### Dietary choline

A cohort study in eastern Finland suggested that higher total choline intake is associated with better cognitive performance [106]. However, recent results from the Framingham Heart Study indicate that high choline intake may increase the risks of dementia and AD, although it did not reach statistical significance [107]. Choline is a primary source of trimethylamine (TMA), a gut microbe-derived metabolite that can be converted to TMAO in the liver. TMAO is a risk factor for age-related cognitive decline and dementia [71]. It is speculated that the composition and function of the gut microbiome may determine whether dietary choline will be converted to TMAO. Further studies are needed to explore the mechanisms underlying this relationship and determine the optimal amount of dietary choline intake.

##### Vitamins

Studies have suggested that vitamins A, B, D and E have neuroprotective effects against AD and PD [108–111]. Importantly, the gut microbiome is involved in vitamin metabolism [112]. Approximately 30% of a person’s daily supply of B group vitamins is from synthesis by certain strains of gut microorganisms [113, 114]. Furthermore, vitamins can directly or indirectly modulate the gut microbiome [115]. A cross-sectional study showed that serum levels of 1,25(OH)_2_D can explain 5% and 2% of variances in α-diversity and β-diversity of the gut microbiome, respectively [116]. Vitamin A deficiency exacerbates learning and memory impairments, increases the Aβ burden in the brain and gut, and downregulates brain-derived neurotrophic factor and GABA receptors in the cortex. These effects are associated with the altered α- and β-diversity of the gut microbiome and a decreased abundance of *Lactobacillus* in AD (APP/PS1) mice [117]. These findings highlight the vital role of the bidirectional interactions between vitamins and the gut microbiome in NDs.

##### Polyunsaturated fatty acids (PUFAs)

Observational and interventional studies have shown that higher levels of PUFAs and long-term PUFA supplementation are associated with a slower cognitive decline in AD patients, as well as reduced motor dysfunctions and a lower incidence of PD [118–122]. Several clinical trials have demonstrated that omega-3 PUFA supplementation can increase the abundance of *Bacteroidetes* and butyrate-producing bacteria belonging to the *Lachnospiraceae* family [123–125]. Importantly, these two taxa are significantly associated with disease severity of NDs [123–125].

In summary, diet plays a crucial role in the onset and development of NDs, and remodelling of the gut microbiome may be one of the possible explanations for this effect. The interactions among diet, gut microbiome and NDs are complex. The current evidence is mainly based on observational results, and further randomized clinical trials (RCTs) are needed to explore their relationships.

### Probiotics and prebiotics

Probiotics and prebiotics are well-known treatments for modulating the gut microbiome and improving human health. Their therapeutic potential in NDs is discussed below.

#### Probiotics

*Lactobacillus* and *Bifidobacterium. Lactobacillus* and *Bifidobacterium* are the most common probiotics and have been widely included in yoghurt and in pharmaceuticals. Tamtaji et al. identified that probiotics (*Lactobacillus acidophilus*, *Bifidobacterium bifidum*, and *Bifidobacterium longum*, 2 × 10^9^ CFU/day) combined with selenium supplementation significantly enhanced cognitive function compared to selenium or placebo alone in AD patients [126]. Administration of probiotic *Lactobacillus* and *Bifidobacterium* strains alleviated both constipation symptoms and motor symptoms in PD patients [127, 128]. However, a meta-analysis that included trials investigating the effects of probiotics, mainly *Lactobacillus* and *Bifidobacterium*, did not find any beneficial effects on cognitive function in diagnosed AD patients [129]. The increased abundance of *Lactobacillus* and *Bifidobacterium* in patients with AD and PD may raise doubts on their potential beneficial effects on NDs. It is important to note that different species within the same genus might have distinct effects. To gain a better understanding, high-quality metagenomic data with strain-level resolution will be instrumental in identifying the specific species of *Lactobacillus* and *Bifidobacterium* that exhibit altered abundance among individuals with NDs. Consequently, further research is necessary to confirm the applicability of *Lactobacillus* and *Bifidobacterium* in NDs.

*Clostridium butyricum.* In addition to these common probiotics, there is a need to explore novel probiotics targeting NDs. *Clostridium butyricum* is a notable probiotic that can ferment carbohydrates into butyric acid and has excellent protective effects against pathogenic microorganisms [130]. An animal study showed that a 4-week treatment with *Clostridium butyricum* (5 × 10^8^ CFU/day) effectively protected against cognitive impairment and amyloid pathology by reducing microglia-mediated neuroinflammation in an AD (APP/PS1) mouse model [131]. Furthermore, *Clostridium butyricum* treatment reverses the abnormal gut microbiome and restores the level of butyrate [131]. *Clostridium butyricum* treatment also ameliorates motor deficits, dopaminergic neuronal loss, synaptic dysfunction and microglial activation in mice with MPTP-induced PD, and these effects are associated with restoration of the gut microbiome and reduced levels of colonic glucagon-like peptide-1 (GLP-1), colonic G-protein-coupled receptor 41/42 and cerebral GLP-1 receptor [132].

*Akkermansia muciniphila.* Ageing is the major risk factor for NDs. Studies in ageing and rejuvenation animal models have shown that *Akkermansia* and the butyrate biosynthesis pathway are crucial in ageing [133]. Preclinical studies showed that *Akkermansia muciniphila* can effectively halt the progression of NDs [134–136]. It significantly reduces the fasting blood glucose and serum diamine oxidase levels, ameliorates the reduction of colonic mucus cells, and further decreases the levels of Aβ40/42 in the cerebral cortex and ameliorates cognitive deficits in APP/PS1 AD mice [134]. Another study revealed that *Akkermansia muciniphila* treatment ameliorates cognitive deficits, Aβ deposition in the brain, and bone loss in AlCl3/D-galactose injection-mediated AD rats [135]. Moreover, it increases the abundance of SCFA-producing or neurotransmitter-producing gut microbiome like *Blautia*, *Staphylococcus*, and *Lactococcus* [135]. *Akkermansia muciniphila* treatment also leads to accumulation of *Akkermansia muciniphila-*associated nicotinamide in the central nervous system, thereby improving motor symptoms and gene expression patterns in the spinal cord of ALS (Sod1-Tg) mice [136]. However, other studies suggest that *Akkermansia* is a mucin-degrading genus that is increased in faecal samples from PD patients and may exacerbate PD progression [137, 138].

More high-quality clinical trials are needed to identify the neuroprotective effects of these specific strains of microorganisms in NDs. The inconsistent and unstable results imply that (1) distinct disease models with different gut microbiome compositions may account for the diverse effects of probiotics; and (2) focusing on the changes in one or two taxa is not enough, as the complex interactions among microbes need to be considered.

#### Prebiotics

Numerous preclinical studies have suggested the potential of prebiotics as an adjuvant treatment for NDs. Intake of soluble fibre for 2 months significantly alleviated astrocyte activation and improved cognitive function in 6-month-old male AD (APP/PS1) mice [139]. These effects depended on the modulation of butyrate/propionate production by gut microbiome, and were eliminated in antibiotic-treated mice, indicating that the neuroprotective effects of soluble fibre depend on the gut microbiome [139]. A 16-week fibre-rich diet ameliorated motor function and α-synuclein aggregation in the substantia nigra of PD (α-synuclein-overexpressing) mice by reversing pathogenic microglial states [140]. The high-fibre diet also improved the cognitive and affective functions as well as the gastrointestinal function of HD mice by altering the gut microbiome profiles [141]. A longitudinal human study showed that higher dietary fructan intake is associated with a lower incidence of AD in the elderly [142]. Furthermore, a diet rich in insoluble fibre significantly improved motor function and constipation symptoms, and increased the plasma level of L-DOPA, in PD patients with constipation [143]. Considering the diversity of the gut microbiome in patients of different sexes, ages and diseases, investigating which prebiotics provide “food” for specific microbes will provide guidance for future applications of prebiotics in specific populations.

### Gut microbiome-derived metabolites

#### SCFAs

Microbiome-derived SCFAs, such as acetate, propionate, and butyrate, are small organic compounds primarily produced from anaerobic fermentation of dietary fibres in the gut and are able to translocate from the gut to the circulation and across the BBB [88]. Sodium butyrate has been observed to enhance the long-term potentiation and depotentiation, and promote the development of dendritic spines and synapse-associated proteins in 5×FAD mice [144]. Wang et al. demonstrated that sodium butyrate has the potential to ameliorate cognitive deficits induced by Aβ_25–35_ injection in mice, which may be attributed to its ability to promote the differentiation of astrocytes towards the A2-neuron-protective subtype [145]. However, the roles of acetate and propionate in AD are controversial. Liu et al. found that microglial activation and cognitive impairment were attenuated by acetate in APP/PS1 mice [146], while another study demonstrated that acetate induced a proinflammatory phenotype in microglia and aggravated Aβ deposition [88]. A recent study showed that propionate treatment suppressed the levels of inducible nitric oxide synthase and pro-inflammatory cytokines and restored synaptic plasticity and cognitive function in Aβ_1−42_-induced AD mice [147]. However, a study in a three-city population-based cohort found that higher serum levels of propionate are associated with increased odds of cognitive decline [148].

In an MPTP-induced PD mouse model, sodium butyrate treatment led to worsening of motor deficits, dopaminergic neuronal loss and glia-mediated neuroinflammation via exacerbating pro-inflammatory cytokine expression and nitric oxide production [149]. However, a study by Sampson et al. revealed that an SCFA mixture caused microglial activation and motor deficits in germ-free α-synuclein overexpressing mice [35]. For ALS, butyrate administration may be beneficial, as it can restore gut microbiome homeostasis, maintain gut integrity, and prolong lifespan in G93A transgenic mouse model of ALS [150]. Overall, the role of SCFAs in NDs is rather complex and may depend on their dosage. Further clinical trials and in-depth basic research are needed to elucidate their roles and mechanisms of action in NDs.

#### Secondary bile acids

Secondary bile acids are gut microbiome-derived molecules that may directly affect NDs. AD individuals show significantly increased serum levels of secondary bile acids, such as deoxycholic acid and its glycine- and taurine-conjugated forms, compared to controls [151]. Higher deoxycholic acid : cholic acid ratio and increased levels of conjugated bile acids (glycodeoxycholic acid, glycolithocholic acid and taurolithocholic acid) are strongly correlated with cognitive decline [151]. In PD patients, the plasma levels of deoxycholic acid and glycodeoxycholic acid are significantly higher than those in controls, and the plasma level of glycodeoxycholic acid is also associated with positive PD status [152]. Furthermore, the plasma level of deoxycholic acid correlates with the relative abundance of *Lachnoclostridium* sp. *An14* and *Anaerotignum lactatifermentans* of the family *Lachnospiraceae*, and the glycodeoxycholic acid concentration correlates with the pro-inflammatory bacterial genera *Alistipes* and *Bacteroides* [152]. The gut microbiome-derived secondary bile acids exert their effects by binding to their receptors, such as farnesoid X receptor, liver X receptor α and β, and sphingosine-1-phosphate receptor 2 [153]. Further investigations of the roles of bile acid receptors may lead to novel therapeutic approaches to treating NDs.

#### TMAO

TMAO has been linked to cell senescence and ND pathogenesis [154, 155]. Inhibiting TMAO formation with 3,3-dimethyl-1-butanol has been shown to ameliorate cognitive dysfunction in APP/PS1 transgenic mice [71, 156, 157]. This suggests that therapeutics aimed at eliminating gut microbiome-derived deleterious metabolites hold promise for treatment of NDs.

#### Other metabolites

Indole-3-propionic acid (IPA) is a gut microbiota metabolite synthesized from tryptophan. Preclinical studies revealed that IPA can improve the intestinal barrier, restore gut microbiome homeostasis and inhibit Aβ fibril formation, suggesting its potential use for the treatment of AD. S-equol, equol, and phenyl-γ‐valerolactones are also promising microbial metabolites for treating NDs [158–163].

### Antibacterials

#### Antibiotics

Antibiotics are widely used to treat infections caused by pathogenic microorganisms, and they also have a significant impact on the gut microbiome. There is growing evidence supporting the “infectious hypothesis” of AD [164, 165]. Hospitalisation for any infectious disease has been found to be associated with a higher risk of AD [164], which implies that the use of antibiotics might be a potential treatment for AD. A study showed that early postnatal antibiotic cocktails (postnatal day 14–21) led to a long-term alteration of gut microbiome composition in the form of increased abundance of *Lachnospiraceae* and *S24-7* and a reduction of Aβ deposition in the brains of aged APP/PS1 mice, with elevated numbers of Foxp3^+^ T regulatory cells in blood and brain [166]. However, administration of broad-spectrum antibiotic cocktails from early adolescence to adulthood exacerbated spatial memory impairment in adult male mice after Aβ_1−42_ microinjection, and decreased TNF-α and IL-6 levels in the brain [167]. These findings imply that the effects of antibiotics can vary depending on the timing, duration, and pharmacological actions of the antibiotics. A clinical trial revealed that both short-term and long-term treatment with D-cycloserine can improve the cognitive functions of AD patients [168]. However, retrospective studies have indicated that broad- and narrow-spectrum antibiotics increase the risks of overall dementia and AD [169, 170]. A multicentre RCT revealed that doxycycline or rifampin treatment resulted in significant deterioration in SADAS-cog score in mild and moderate AD patients [171].

Antibiotics have also been found to significantly improve motor dysfunction and alleviate neuroinflammation by increasing the relative abundance of *Akkermansia* and *Lachnospiraceae* and SCFA levels in the faeces of MPTP-induced and transgenic PD mouse models [172–174]. A 12-month study found that creatine and minocycline can delay the disease progression in PD patients [175]. However, a nationwide case-control study in Finland showed a positive association between exposure to certain antibiotics (including antianaerobics, tetracyclines, sulfonamides and trimethoprim) and an increased incidence of PD [176]. A nested case-control study conducted in Sweden revealed that the use of any antibiotics is associated with a higher risk of ALS, particularly for those who have received more than two prescriptions of beta-lactamase sensitive penicillin [177].

While there is promising evidence from preclinical studies about the potential role of antibiotics in preventing NDs, more research is needed to translate these findings into effective treatments in patients. The gut microbiome is a complex ecosystem with many microorganisms, and it is challenging to inhibit or promote the growth of specific microbes. Moreover, the widespread use of antibiotics can lead to the emergence of drug-resistant bacteria. Therefore, it is necessary to explore solutions to address the limitations of antibiotics.

#### Bacteriophages

Currently, nonbacterial constituents of the microbiome, such as viruses, have received much attention. Bacteriophages are viruses that infect bacteria and have been widely used as antibacterial agents to modulate the gut microbiome and treat diseases for over 100 years [178, 179]. A recent preclinical study revealed that *Ruminococcaceae* and its metabolite isoamylamine are highly enriched in aged mice and elderly people, contributing to neuroinflammation and age-related cognitive decline [180]. Isoamylamine can aggravate apoptosis of microglia by recruiting the transcriptional regulator p53 to the S100A8 promoter region, leading to microglial dysfunction [180]. Interestingly, the presence of *Myoviridae* bacteriophage, which targets *Ruminococcaceae*, is significantly reduced in the gut of aged mice [180]. Oral administration of *Myoviridae* bacteriophage dramatically decreases the level of isoamylamine in the gut and reduces neuroinflammation [180]. These findings suggest that bacteriophages may be powerful tools for modulating the gut microbiome. More preclinical and clinical studies are needed to determine the therapeutic potential of bacteriophages for NDs.

### FMT and synthetic microbiome transplantation

#### FMT

FMT is an emerging potent method for restoring the gut ecosystem. The species-rich microbiomes as well as their products and metabolites from healthy donors can increase the microbial diversity, alter the production of metabolites from certain microbes and the host, and alter the host immune response [181]. Studies in mouse models have shown that transplantation of the faecal microbiome from healthy (wild-type) mice with antibiotic cocktail pretreatment can reverse the abnormalities in the colonic expression of genes related to gut macrophage activity and the circulating inflammatory monocytes, and subsequently reduce brain Aβ burden and phosphorylation of tau protein and improve the cognitive function of AD (ADLP^APT^) mice [59, 182]. Additionally, another study demonstrated that FMT from young or healthy (wild-type) mice, in the absence of antibiotic administration, can also ameliorate Aβ plaques and cognitive impairments in 5×FAD mice [183]. In rotenone-induced PD mice, motor deficits were ameliorated two weeks after FMT from healthy mice, without pre-antibiotic treatment. These protective effects might be attributed to the reductions in LPS and the TLR4/myeloid differentiation primary response gene 88/ nuclear factor-kappa B signalling pathway in the colon, serum and brain, along with improved BBB integrity [184]. A few clinical cases or trials investigating the effects of FMT in AD and PD patients have shown promising results. For instance, at 48 h after discontinuation of antibiotic prescriptions (vancomycin and metronidazole), dementia patients with cognitive decline and *Clostridioides difficile* infection received FMT via colonoscopy [185]. They showed enhanced cognitive function one month after receiving FMT from healthy donors, compared to the baseline [185]. Recently, Cheng et al. enrolled 54 PD patients, with 27 receiving oral FMT capsules and the remaining 27 receiving placebo capsules without antibiotics pretreatment [186]. Patients who underwent oral FMT showed improvement in PD-related autonomic symptoms, gastrointestinal disorders and the complexity of the microecological system at three months post-FMT [186]. Another study included 15 PD patients, 10 receiving colonic FMT and 5 nasointestinal FMT [187]. FMT relieved the motor deficits, improved the quality of life and sleep, and relieved anxiety and depression at 1 and 3 months post-FMT, although 5 of these 15 patients reported adverse events including diarrhoea, flatulence and abdominal pain [187]. In addition, the colonic FMT was more effective than the nasointestinal FMT and maintained the efficacy for a longer time [187].

While FMT shows promise as a therapeutic approach for AD and PD, there are still many questions to be answered. (1) Of the various FMT approaches, including upper gastrointestinal (i.e., capsule delivery, nasointestinal tubes) and lower gastrointestinal routes (i.e., retention enema, sigmoidoscopy or colonoscopy), which one is more suitable for NDs [188]? (2) What are the optimal duration and timing of FMT for NDs? (3) It is unknown whether antibiotic pretreatment is necessary. (4) The quality of donor faeces is a determinant for the effectiveness of FMT, which limits the standard implementation of FMT in large populations.

#### Synthetic microbiome transplantation

Although traditional FMT is relatively safe, the donor faecal microbiome is like a “black box” that contains many unknown ingredients, such as bacteria, yeasts, parasites and viruses, which can be detrimental for recipients. Recently, researchers have proposed the “synthetic microbiome” for transplantation, which has a well-defined composition of bacteria. Cheng et al. designed a community of 104 bacterial species called “hCom2”. Mice colonized by the hCom2 had a similar phenotype as those colonized by the human faecal community [189]. This concept suggests that designing a synthetic microbiome mimicking the gut microbiome of healthy individuals and adding specific probiotics that are beneficial for patients with NDs may be a promising strategy to overcome the shortcomings of traditional FMT in treating NDs.

## Perspectives and conclusion

Intriguing preclinical and clinical evidence reveals the crucial role of the gut microbiome in the development of NDs. Modulation of the gut microbiome has emerged as a potential therapeutic option outside of the brain. However, there is a long way to go from preclinical or observational evidence to clinical application.

Although there is a general consensus that the gut microbiome is significantly altered in NDs and can modify the risks and progression of NDs, much remains unknown. Defining the characteristics of the gut microbiome in NDs and identifying precise markers to differentiate between ND patients and healthy individuals is paramount. High-quality metagenomic data with strain-level resolution and metabolomics data across large longitudinal cohort studies will help address these challenges. Moreover, clear mechanistic details concerning the routes through which gut bacteria and their products impact the brain are currently unknown. Further research into how gut microbes modulate immune cells, regulate amyloid deposition and tau aggregation in the gut and the brain, and pass through barriers may lead to development of drugs and therapeutic strategies that target the gut microbiome.

In the present review, we discussed various potential clinical approaches aimed at targeting the gut microbiome in NDs. Among these, probiotics, antibacterials and FMT directly modify the composition of the gut microbiome. In contrast, dietary patterns, nutritional supplements, and prebiotics supply “nutritional substances” and create a modified living environment for the gut microbiome, thereby indirectly regulating its profile. Additionally, the supplementation of beneficial metabolites and the elimination of harmful metabolites focus on the “secondary products” of the gut microbiome. All of these gut microbiome-targeted strategies have proven effective for NDs. However, the mechanisms underlying these promising therapeutic approaches for NDs remain largely unknown, necessitating further in-depth exploration.

The host–microbiome interactions in NDs are complex. The gut microbial DNA expression and pathways are regulated by molecules produced by the host gut epithelial cells [190]. Studies have identified *APOE* ε4 and rs429358 alleles as risk factors for AD, which show a positive correlation with the abundance of pathogenic *Proteobacteria* and the proinflammatory genus *Collinsella*, respectively [191, 192]. In addition, the A53T transgenic monkeys show α-synuclein aggregation in the gut, a higher degree of diversity of the gut microbiome, and elevated abundance of *Sybergistetes*, *Akkermansia*, and *Eggerthella lenta* [193]. Building on the above evidence regarding the potential of gut microbiome modulation to halt the progression of NDs, it is speculated that targeting the gut microbiome could be an effective therapeutic approach for patients susceptible to NDs due to genetic factors. Moreover, gut microbiome treatments should be tailored based on the unique microbial alterations observed in different ND patients.

Considering that the gut microbiome ecosystem is complex and has variability among individuals and that the equilibrium of the ecosystem is maintained by microbial diversity and stability as well as microbe–microbe and host–microbe interactions [194], the final goal of microbial modulation is the maintenance of equilibrium of the gut microbiome ecosystem based on an individual’s microbiome. Standardization and industrialization of methods for engineered probiotics, gut-derived metabolite analogues, synthetic human gut microbiomes for FMT, etc., are urgently needed. The gut microbiome is a promising approach for understanding and treating NDs, so future studies should explore the in-depth mechanisms involved in the gut microbiome–brain axis to identify the optimal ways to maintain and restore the equilibrium of the gut microbiome ecosystem.

## Data Availability

Not applicable.

## Abbreviations

Aβ: Amyloid-β
AD: Alzheimer’s disease
ALS: Amyotrophic lateral sclerosis
FMT: Faecal microbiome transplantation
GABA: γ-Aminobutyric acid
GLP-1: Glucagon-like peptide-1
HD: Huntington’s disease
HDGECs: HD gene expansion carriers
IL: Interleukin
IPA: Indole-3-propionic acid
LPS: Lipopolysaccharide
MeDi: Mediterranean
MIND: Mediterranean-DASH diet intervention for neurodegenerative delay
ND: Neurodegenerative disease
PUFA: Polyunsaturated fatty acid
RBD: REM sleep behaviour disorder
RBD-FDR: First-degree relatives of RBD
SCFA: Short-chain fatty acid
SPF: Specific pathogen-free
TLR4: Toll-like receptor 4
TMA: Trimethylamine
TMAO: Trimethylamine-*N*‐oxide
PD: Parkinson’s disease

## References

[1] Ramanan VK, Saykin AJ (2013). Pathways to neurodegeneration: mechanistic insights from GWAS in Alzheimer’s disease, Parkinson’s disease, and related disorders. Am J Neurodegener Dis.

[2] Zhu Z, Yang Z, Yu L, Xu L, Wu Y, Zhang X (2023). Residential greenness, air pollution and incident neurodegenerative disease: a cohort study in China. Sci Total Environ.

[3] Wang J, Gu BJ, Masters CL, Wang Y-J (2017). A systemic view of Alzheimer disease—insights from amyloid-β metabolism beyond the brain. Nat Rev Neurol.

[4] Farrow SL, Cooper AA, O’Sullivan JM (2022). Redefining the hypotheses driving Parkinson’s diseases research. NPJ Parkinsons Dis.

[5] Ritz NL, Brocka M, Butler MI, Cowan CSM, Barrera-Bugueño C, Turkington CJR (2023). Social anxiety disorder-associated gut microbiota increases social fear. Proc Natl Acad Sci USA.

[6] Ross FC, Mayer DE, Gupta A, Gill CIR, Del Rio D, Cryan JF (2024). Existing and future strategies to manipulate the gut microbiota with diet as a potential adjuvant treatment for psychiatric disorders. Biol Psychiatry.

[7] Ridaura V, Belkaid Y (2015). Gut microbiota: the link to your second brain. Cell.

[8] Brown K, Thomson CA, Wacker S, Drikic M, Groves R, Fan V (2023). Microbiota alters the metabolome in an age- and sex- dependent manner in mice. Nat Commun.

[9] Bianchimano P, Britton GJ, Wallach DS, Smith EM, Cox LM, Liu S (2022). Mining the microbiota to identify gut commensals modulating neuroinflammation in a mouse model of multiple sclerosis. Microbiome.

[10] Shandilya S, Kumar S, Kumar Jha N, Kumar Kesari K, Ruokolainen J (2022). Interplay of gut microbiota and oxidative stress: perspective on neurodegeneration and neuroprotection. J Adv Res.

[11] Zhang Y, Shen Y, Liufu N, Liu L, Li W, Shi Z (2023). Transmission of Alzheimer’s disease-associated microbiota dysbiosis and its impact on cognitive function: evidence from mice and patients. Mol Psychiatry.

[12] Zhang X, Tang B, Guo J (2023). Parkinson’s disease and gut microbiota: from clinical to mechanistic and therapeutic studies. Transl Neurodegener.

[13] Zhang T, Gao G, Kwok L-Y, Sun Z (2023). Gut microbiome-targeted therapies for Alzheimer’s disease. Gut Microbes.

[14] Molinero N, Antón-Fernández A, Hernández F, Ávila J, Bartolomé B, Moreno-Arribas MV (2023). Gut microbiota, an additional hallmark of human aging and neurodegeneration. Neuroscience.

[15] Liu L, Huh JR, Shah K (2022). Microbiota and the gut-brain-axis: implications for new therapeutic design in the CNS. eBioMedicine.

[16] Hashim HM, Makpol S (2022). A review of the preclinical and clinical studies on the role of the gut microbiome in aging and neurodegenerative diseases and its modulation. Front Cell Neurosci.

[17] Wu JH, Guo Z, Kumar S, Lapuerta P (2011). Incidence of serious upper and lower gastrointestinal events in older adults with and without Alzheimer’s disease. J Am Geriatr Soc.

[18] Warnecke T, Schäfer KH, Claus I, Del Tredici K, Jost WH (2022). Gastrointestinal involvement in Parkinson’s disease: pathophysiology, diagnosis, and management. NPJ Parkinsons Dis.

[19] Parra-Cantu C, Zaldivar-Ruenes A, Martinez-Vazquez M, Martinez HR (2021). Prevalence of gastrointestinal symptoms, severity of dysphagia, and their correlation with severity of amyotrophic lateral sclerosis in a Mexican cohort. Neurodegener Dis.

[20] Wronka D, Karlik A, Misiorek JO, Przybyl L (2023). What the gut tells the brain—Is there a link between microbiota and Huntington’s disease?. Int J Mol Sci.

[21] Fang P, Kazmi SA, Jameson KG, Hsiao EY (2020). The microbiome as a modifier of neurodegenerative disease risk. Cell Host Microbe.

[22] Wasser CI, Mercieca E-C, Kong G, Hannan AJ, McKeown SJ, Glikmann-Johnston Y (2020). Gut dysbiosis in Huntington’s disease: associations among gut microbiota, cognitive performance and clinical outcomes. Brain Commun.

[23] Hung C-C, Chang C-C, Huang C-W, Nouchi R, Cheng C-H (2022). Gut microbiota in patients with Alzheimer’s disease spectrum: a systematic review and meta-analysis. Aging.

[24] Sheng C, Yang K, He B, Du W, Cai Y, Han Y (2022). Combination of gut microbiota and plasma amyloid-β as a potential index for identifying preclinical Alzheimer’s disease: a cross-sectional analysis from the SILCODE study. Alzheimers Res Ther.

[25] Ferreiro AL, Choi J, Ryou J, Newcomer EP, Thompson R, Bollinger RM (2023). Gut microbiome composition may be an indicator of preclinical Alzheimer’s disease. Sci Transl Med.

[26] Qian X, Liu X, Chen G, Chen S, Tang H (2022). injection of amyloid-β to lateral ventricle induces gut microbiota dysbiosis in association with inhibition of cholinergic anti-inflammatory pathways in Alzheimer’s disease. J Neuroinflamm.

[27] Kim N, Jeon SH, Ju IG, Gee MS, Do J, Oh MS (2021). Transplantation of gut microbiota derived from Alzheimer’s disease mouse model impairs memory function and neurogenesis in C57BL/6 mice. Brain Behav Immun.

[28] Chen C, Liao J, Xia Y, Liu X, Jones R, Haran J (2022). Gut microbiota regulate Alzheimer’s disease pathologies and cognitive disorders via PUFA-associated neuroinflammation. Gut.

[29] Dodiya HB, Lutz HL, Weigle IQ, Patel P, Michalkiewicz J, Roman-Santiago CJ (2022). Gut microbiota-driven brain Aβ amyloidosis in mice requires microglia. J Exp Med.

[30] Harach T, Marungruang N, Duthilleul N, Cheatham V, Mc Coy KD, Frisoni G (2017). Reduction of abeta amyloid pathology in APPPS1 transgenic mice in the absence of gut microbiota. Sci Rep.

[31] Seo D-o, O’Donnell D, Jain N, Ulrich JD, Herz J, Li Y (2023). APOE isoform- and microbiota-dependent progression of neurodegeneration in a mouse model of tauopathy. Science.

[32] Tan AH, Lim SY, Lang AE (2022). The microbiome–gut–brain axis in Parkinson disease—from basic research to the clinic. Nat Rev Neurol.

[33] Wang Q, Luo Y, Ray Chaudhuri K, Reynolds R, Tan E-K, Pettersson S (2021). The role of gut dysbiosis in Parkinson’s disease: mechanistic insights and therapeutic options. Brain.

[34] Huang B, Chau SWH, Liu Y, Chan JWY, Wang J, Ma SL (2023). Gut microbiome dysbiosis across early Parkinson’s disease, REM sleep behavior disorder and their first-degree relatives. Nat Commun.

[35] Sampson TR, Debelius JW, Thron T, Janssen S, Shastri GG, Ilhan ZE (2016). Gut microbiota regulate motor deficits and neuroinflammation in a model of Parkinson’s disease. Cell.

[36] Martin S, Battistini C, Sun J (2022). A gut feeling in amyotrophic lateral sclerosis: Microbiome of mice and men. Front Cell Infect Microbiol.

[37] Gong Z, Ba L, Tang J, Yang Y, Li Z, Liu M (2023). Gut microbiota links with cognitive impairment in amyotrophic lateral sclerosis: a multi-omics study. J Biomed Res.

[38] Cox LM, Calcagno N, Gauthier C, Madore C, Butovsky O, Weiner HL (2022). The microbiota restrains neurodegenerative microglia in a model of amyotrophic lateral sclerosis. Microbiome.

[39] Du G, Dong W, Yang Q, Yu X, Ma J, Gu W (2021). Altered gut microbiota related to inflammatory responses in patients with Huntington’s disease. Front Immunol.

[40] Gubert C, Choo JM, Love CJ, Kodikara S, Masson BA, Liew JJM (2022). Faecal microbiota transplant ameliorates gut dysbiosis and cognitive deficits in Huntington’s disease mice. Brain Commun.

[41] Liang L, Yue Y, Zhong L, Liang Y, Shi R, Luo R (2023). Anti-aging activities of Rehmannia Glutinosa Libosch. Crude polysaccharide in *Caenorhabditis elegans* based on gut microbiota and metabonomic analysis. Int J Biol Macromol.

[42] Zeng X, Li X, Li X, Wei C, Shi C, Hu K (2023). Fecal microbiota transplantation from young mice rejuvenates aged hematopoietic stem cells by suppressing inflammation. Blood.

[43] Gai X, Wang H, Li Y, Zhao H, He C, Wang Z (2021). Fecal microbiota transplantation protects the intestinal mucosal barrier by reconstructing the gut microbiota in a murine model of sepsis. Front Cell Infect Microbiol.

[44] Yang C-J, Chang H-C, Sung P-C, Ge M-C, Tang H-Y, Cheng M-L (2024). Oral fecal transplantation enriches Lachnospiraceae and butyrate to mitigate acute liver injury. Cell Rep.

[45] Loffredo L, Ettorre E, Zicari AM, Inghilleri M, Nocella C, Perri L et al. Oxidative stress and gut-derived lipopolysaccharides in neurodegenerative disease: Role of NOX2. Oxid Med Cell Longev. 2020; 2020:1–7. 10.1155/2020/8630275 PMC7016401 32089785

[46] Das TK, Ganesh BP (2023). Interlink between the gut microbiota and inflammation in the context of oxidative stress in Alzheimer’s disease progression. Gut Microbes.

[47] DeMaio A, Mehrotra S, Sambamurti K, Husain S (2022). The role of the adaptive immune system and T cell dysfunction in neurodegenerative diseases. J Neuroinflamm.

[48] Harms AS, Ferreira SA, Romero-Ramos M (2021). Periphery and brain, innate and adaptive immunity in Parkinson’s disease. Acta Neuropathol.

[49] Yu W, He J, Cai X, Yu Z, Zou Z, Fan D (2022). Neuroimmune crosstalk between the peripheral and the central immune system in amyotrophic lateral sclerosis. Front Aging Neurosci.

[50] Malvaso A, Gatti A, Negro G, Calatozzolo C, Medici V, Poloni TE (2023). Microglial senescence and activation in healthy aging and Alzheimer’s disease: systematic review and neuropathological scoring. Cells.

[51] Han T, Xu Y, Sun L, Hashimoto M, Wei J (2024). Microglial response to aging and neuroinflammation in the development of neurodegenerative diseases. Neural Regen Res.

[52] Khosravi A, Yáñez A, Price Jeremy G, Chow A, Merad M, Goodridge Helen S (2014). Gut microbiota promote hematopoiesis to control bacterial infection. Cell Host Microbe.

[53] Erny D, Hrabě de Angelis AL, Jaitin D, Wieghofer P, Staszewski O, David E (2015). Host microbiota constantly control maturation and function of microglia in the CNS. Nat Neurosci.

[54] Thion MS, Low D, Silvin A, Chen J, Grisel P, Schulte-Schrepping J (2018). Microbiome influences prenatal and adult microglia in a sex-specific manner. Cell.

[55] Mossad O, Batut B, Yilmaz B, Dokalis N, Mezö C, Nent E (2022). Gut microbiota drives age-related oxidative stress and mitochondrial damage in microglia via the metabolite N^6^-carboxymethyllysine. Nat Neurosci.

[56] Kolypetri P, Liu S, Cox LM, Fujiwara M, Raheja R, Ghitza D (2021). Regulation of splenic monocyte homeostasis and function by gut microbial products. iScience.

[57] Hergott CB, Roche AM, Tamashiro E, Clarke TB, Bailey AG, Laughlin A (2016). Peptidoglycan from the gut microbiota governs the lifespan of circulating phagocytes at homeostasis. Blood.

[58] Kolypetri P, Weiner HL (2023). Monocyte regulation by gut microbial signals. Trends Microbiol.

[59] Kim M-S, Kim Y, Choi H, Kim W, Park S, Lee D (2020). Transfer of a healthy microbiota reduces amyloid and tau pathology in an Alzheimer’s disease animal model. Gut.

[60] Xu Y, Li Y, Wang C, Han T, Liu H, Sun L (2023). The reciprocal interactions between microglia and T cells in Parkinson’s disease: a double-edged sword. J Neuroinflamm.

[61] Liu Z, Cheng X, Zhong S, Zhang X, Liu C, Liu F (2020). Peripheral and central nervous system immune response crosstalk in amyotrophic lateral sclerosis. Front Neurosci.

[62] Gericke C, Kirabali T, Flury R, Mallone A, Rickenbach C, Kulic L (2023). Early β-amyloid accumulation in the brain is associated with peripheral T cell alterations. Alzheimers Dement.

[63] Chen X, Firulyova M, Manis M, Herz J, Smirnov I, Aladyeva E (2023). Microglia-mediated T cell infiltration drives neurodegeneration in tauopathy. Nature.

[64] Pasciuto E, Burton OT, Roca CP, Lagou V, Rajan WD, Theys T (2020). Microglia require CD4 T cells to complete the fetal-to-adult transition. Cell.

[65] Bai X-B, Xu S, Zhou L-J, Meng X-Q, Li Y-L, Chen Y-L (2023). Oral pathogens exacerbate Parkinson’s disease by promoting Th1 cell infiltration in mice. Microbiome.

[66] Rei D, Saha S, Haddad M, Rubio AH, Perlaza BL, Berard M (2022). Age-associated gut microbiota impair hippocampus-dependent memory in a vagus-dependent manner. JCI Insight.

[67] Vargas-Caballero M, Warming H, Walker R, Holmes C, Cruickshank G, Patel B (2022). Vagus nerve stimulation as a potential therapy in early Alzheimer’s disease: a review. Front Hum Neurosci.

[68] Torrecillos F, Tan H, Brown P, Capone F, Ricciuti R, Di Lazzaro V (2022). Non-invasive vagus nerve stimulation modulates subthalamic beta activity in Parkinson’s disease. Brain Stimulat.

[69] Yu Y, Jiang X, Fang X, Wang Y, Liu P, Ling J (2023). Transauricular vagal nerve stimulation at 40 hz inhibits hippocampal P2 × 7R/NLRP3/Caspase-1 signaling and improves spatial learning and memory in 6-month-old APP/PS1 mice. Neuromodulation.

[70] Wu Y, Zhang Y, Xie B, Abdelgawad A, Chen X, Han M (2021). Rhanp attenuates endotoxin-derived cognitive dysfunction through subdiaphragmatic vagus nerve-mediated gut microbiota–brain axis. J Neuroinflammation.

[71] Connell E, Le Gall G, Pontifex MG, Sami S, Cryan JF, Clarke G (2022). Microbial-derived metabolites as a risk factor of age-related cognitive decline and dementia. Mol Neurodegener.

[72] Joachim CL, Mori H, Selkoe DJ (1989). Amyloid beta-protein deposition in tissues other than brain in Alzheimer’s disease. Nature.

[73] Ahn EH, Kang SS, Liu X, Chen G, Zhang Z, Chandrasekharan B (2019). Initiation of Parkinson’s disease from gut to brain by δ-secretase. Cell Res.

[74] Wang R, Ren H, Kaznacheyeva E, Lu X, Wang G (2022). Association of glial activation and α-synuclein pathology in Parkinson’s disease. Neurosci Bull.

[75] Bliska JB, Friedland RP, Chapman MR (2017). The role of microbial amyloid in neurodegeneration. PLoS Pathog.

[76] Sun Y, Sommerville NR, Liu JYH, Ngan MP, Poon D, Ponomarev ED (2020). Intra-gastrointestinal amyloid‐β1–42 oligomers perturb enteric function and induce Alzheimer’s disease pathology. J Physiol.

[77] Chandra R, Sokratian A, Chavez KR, King S, Swain SM, Snyder JC (2023). Gut mucosal cells transfer α-synuclein to the vagus nerve. JCI Insight.

[78] Sun B, Sawant H, Borthakur A, Bihl JC (2023). Emerging therapeutic role of gut microbial extracellular vesicles in neurological disorders. Front Neurosci.

[79] Lee K-E, Kim J-K, Han S-K, Lee DY, Lee H-J, Yim S-V (2020). The extracellular vesicle of gut microbial paenalcaligenes hominis is a risk factor for vagus nerve-mediated cognitive impairment. Microbiome.

[80] Svensson E, Horváth-Puhó E, Thomsen RW, Djurhuus JC, Pedersen L, Borghammer P (2015). Vagotomy and subsequent risk of Parkinson’s disease. Ann Neurol.

[81] Liu B, Fang F, Pedersen NL, Tillander A, Ludvigsson JF, Ekbom A (2017). Vagotomy and Parkinson disease. Neurology.

[82] Potgieter M, Bester J, Kell DB, Pretorius E, Danchin PA (2015). The dormant blood microbiome in chronic, inflammatory diseases. FEMS Microbiol Rev.

[83] Li B, He Y, Ma J, Huang P, Du J, Cao L (2019). Mild cognitive impairment has similar alterations as Alzheimer’s disease in gut microbiota. Alzheimers Dement.

[84] Oreja-Guevara C, Forsyth CB, Shannon KM, Kordower JH, Voigt RM, Shaikh M (2011). Increased intestinal permeability correlates with sigmoid mucosa alpha-synuclein staining and endotoxin exposure markers in early Parkinson’s disease. PLoS ONE.

[85] Marizzoni M, Cattaneo A, Mirabelli P, Festari C, Lopizzo N, Nicolosi V (2020). Short-chain fatty acids and lipopolysaccharide as mediators between gut dysbiosis and amyloid pathology in Alzheimer’s disease. J Alzheimers Dis.

[86] Zhao Y, Jaber VR, Pogue AI, Sharfman NM, Taylor C, Lukiw WJ (2022). Lipopolysaccharides (LPSs) as potent neurotoxic glycolipids in Alzheimer’s disease (AD). Int J Mol Sci.

[87] Moné Y, Earl JP, Król JE, Ahmed A, Sen B, Ehrlich GD (2023). Evidence supportive of a bacterial component in the etiology for Alzheimer’s disease and for a temporal-spatial development of a pathogenic microbiome in the brain. Front Cell Infect Microbiol.

[88] Erny D, Dokalis N, Mezö C, Castoldi A, Mossad O, Staszewski O (2021). Microbiota-derived acetate enables the metabolic fitness of the brain innate immune system during health and disease. Cell Metab.

[89] Qiao C-M, Quan W, Zhou Y, Niu G-Y, Hong H, Wu J (2023). Orally induced high serum level of trimethylamine N-oxide worsened glial reaction and neuroinflammation on MPTP-induced acute Parkinson’s disease model mice. Mol Neurobiol.

[90] Zhang Y, Jian W (2023). Signal pathways and intestinal flora through trimethylamine N-oxide in Alzheimer’s disease. Curr Protein Pept Sci.

[91] Glans I, Sonestedt E, Nägga K, Gustavsson A-M, González-Padilla E, Borne Y (2023). Association between dietary habits in midlife with dementia incidence over a 20-year period. Neurology.

[92] Molsberry S, Bjornevik K, Hughes KC, Healy B, Schwarzschild M, Ascherio A (2020). Diet pattern and prodromal features of Parkinson disease. Neurology.

[93] Shivappa N, Steck SE, Hurley TG, Hussey JR, Hébert JR (2013). Designing and developing a literature-derived, population-based dietary inflammatory index. Public Health Nutr.

[94] Balomenos V, Bounou L, Charisis S, Stamelou M, Ntanasi E, Georgiadi K (2022). Dietary inflammatory index score and prodromal Parkinson’s disease incidence: the HELIAD study. J Nutr Biochem.

[95] Charisis S, Ntanasi E, Yannakoulia M, Anastasiou CA, Kosmidis MH, Dardiotis E (2021). Diet inflammatory index and dementia incidence. Neurology.

[96] Zheng J, Hoffman KL, Chen J-S, Shivappa N, Sood A, Browman GJ (2020). Dietary inflammatory potential in relation to the gut microbiome: results from a cross-sectional study. Br J Nutr.

[97] Metcalfe-Roach A, Yu AC, Golz E, Cirstea M, Sundvick K, Kliger D (2021). Mind and Mediterranean diets associated with later onset of Parkinson’s disease. Mov Disord.

[98] Moustafa B, Trifan G, Isasi CR, Lipton RB, Sotres-Alvarez D, Cai J (2022). Association of Mediterranean diet with cognitive decline among diverse hispanic or latino adults from the Hispanic Community Health Study/Study of Latinos. JAMA Netw Open.

[99] de la Rubia Ortí JE, García-Pardo MP, Drehmer E, Sancho Cantus D, Julián Rochina M, Aguilar MA (2018). Improvement of main cognitive functions in patients with Alzheimer’s disease after treatment with coconut oil enriched Mediterranean diet: a pilot study. J Alzheimers Dis.

[100] Paknahad Z, Sheklabadi E, Derakhshan Y, Bagherniya M, Chitsaz A (2020). The effect of the Mediterranean diet on cognitive function in patients with Parkinson’s disease: a randomized clinical controlled trial. Complement Ther Med.

[101] Hoscheidt S, Sanderlin AH, Baker LD, Jung Y, Lockhart S, Kellar D (2021). Mediterranean and western diet effects on Alzheimer’s disease biomarkers, cerebral perfusion, and cognition in mid-life: a randomized trial. Alzheimers Dement.

[102] Solch RJ, Aigbogun JO, Voyiadjis AG, Talkington GM, Darensbourg RM, O’Connell S (2022). Mediterranean diet adherence, gut microbiota, and Alzheimer’s or Parkinson’s disease risk: a systematic review. J Neurol Sci.

[103] Phillips MCL, Deprez LM, Mortimer GMN, Murtagh DKJ, McCoy S, Mylchreest R (2021). Randomized crossover trial of a modified ketogenic diet in Alzheimer’s disease. Alzheimers Res Ther.

[104] Phillips MCL, Murtagh DKJ, Gilbertson LJ, Asztely FJS, Lynch CDP (2018). Low-fat versus ketogenic diet in Parkinson’s disease: a pilot randomized controlled trial. Mov Disord.

[105] Olson CA, Vuong HE, Yano JM, Liang QY, Nusbaum DJ, Hsiao EY (2018). The gut microbiota mediates the anti-seizure effects of the ketogenic diet. Cell.

[106] Ylilauri MPT, Voutilainen S, Lönnroos E, Virtanen HEK, Tuomainen T-P, Salonen JT (2019). Associations of dietary choline intake with risk of incident dementia and with cognitive performance: the Kuopio Ischaemic Heart Disease risk factor study. Am J Clin Nutr.

[107] Yuan J, Liu X, Liu C, Ang AFA, Massaro J, Devine SA (2022). Is dietary choline intake related to dementia and Alzheimer’s disease risks? Results from the Framingham Heart Study. Am J Clin Nutr.

[108] Gong X, Shi L, Wu Y, Luo Y, Kwok T (2022). B vitamin supplementation slows cognitive decline in mild cognitive impairment patients with frontal lobe atrophy. J Alzheimers Dis.

[109] Dysken MW, Sano M, Asthana S, Vertrees JE, Pallaki M, Llorente M (2014). Effect of vitamin E and memantine on functional decline in Alzheimer disease. JAMA.

[110] de Lau LML, Koudstaal PJ, Witteman JCM, Hofman A, Breteler MMB (2006). Dietary folate, vitamin B 12, and vitamin B 6 and the risk of Parkinson disease. Neurology.

[111] Thiel A, Hermanns C, Lauer AA, Reichrath J, Erhardt T, Hartmann T (2023). Vitamin D and its analogues: from differences in molecular mechanisms to potential benefits of adapted use in the treatment of Alzheimer’s disease. Nutrients.

[112] Brauer-Nikonow A, Zimmermann M (2022). How the gut microbiota helps keep us vitaminized. Cell Host Microbe.

[113] LeBlanc JG, Milani C, de Giori GS, Sesma F, van Sinderen D, Ventura M (2013). Bacteria as vitamin suppliers to their host: a gut microbiota perspective. Curr Opin Biotechnol.

[114] Jiang Q, Lin L, Xie F, Jin W, Zhu W, Wang M (2022). Metagenomic insights into the microbe-mediated B and K2 vitamin biosynthesis in the gastrointestinal microbiome of ruminants. Microbiome.

[115] Jiang S, Zhu Q, Mai M, Yang W, Du G (2020). Vitamin B and vitamin D as modulators of gut microbiota in overweight individuals. Int J Food Sci Nutr.

[116] Thomas RL, Jiang L, Adams JS, Xu ZZ, Shen J, Janssen S (2020). Vitamin D metabolites and the gut microbiome in older men. Nat Commun.

[117] Chen B-W, Zhang K-W, Chen S-J, Yang C, Li P-G (2021). Vitamin A deficiency exacerbates gut microbiota dysbiosis and cognitive deficits in amyloid precursor protein/presenilin 1 transgenic mice. Front Aging Neurosci.

[118] Chu C-S, Hung C-F, Ponnusamy VK, Chen K-C, Chen N-C (2022). Higher serum dha and slower cognitive decline in patients with Alzheimer’s disease: two-year follow-up. Nutrients.

[119] Avallone R, Vitale G, Bertolotti M (2019). Omega-3 fatty acids and neurodegenerative diseases: new evidence in clinical trials. Int J Mol Sci.

[120] Chiu C-C, Su K-P, Cheng T-C, Liu H-C, Chang C-J, Dewey ME (2008). The effects of omega-3 fatty acids monotherapy in Alzheimer’s disease and mild cognitive impairment: a preliminary randomized double-blind placebo-controlled study. Prog Neuropsychopharmacol Biol Psychiatry.

[121] da Silva TM, Munhoz RP, Alvarez C, Naliwaiko K, Kiss Á, Andreatini R (2008). Depression in Parkinson’s disease: a double-blind, randomized, placebo-controlled pilot study of omega-3 fatty-acid supplementation. J Affect Disord.

[122] Zhang Y-P, Miao R, Li Q, Wu T, Ma F (2016). Effects of DHA supplementation on hippocampal volume and cognitive function in older adults with mild cognitive impairment: a 12-month randomized, double-blind, placebo-controlled trial. J Alzheimers Dis.

[123] Zhuang Z-Q, Shen L-L, Li W-W, Fu X, Zeng F, Gui L (2018). Gut microbiota is altered in patients with Alzheimer’s disease. J Alzheimers Dis.

[124] Costantini L, Molinari R, Farinon B, Merendino N (2017). Impact of omega-3 fatty acids on the gut microbiota. Int J Mol Sci.

[125] Zheng S-Y, Li H-X, Xu R-C, Miao W-T, Dai M-Y, Ding S-T (2021). Potential roles of gut microbiota and microbial metabolites in Parkinson’s disease. Ageing Res Rev.

[126] Tamtaji OR, Heidari-soureshjani R, Mirhosseini N, Kouchaki E, Bahmani F, Aghadavod E (2019). Probiotic and selenium co-supplementation, and the effects on clinical, metabolic and genetic status in Alzheimer’s disease: a randomized, double-blind, controlled trial. Clin Nutr.

[127] Hong C-T, Chen J-H, Huang T-W (2022). Probiotics treatment for Parkinson disease: a systematic review and meta-analysis of clinical trials. Aging.

[128] Tamtaji OR, Taghizadeh M, Daneshvar Kakhaki R, Kouchaki E, Bahmani F, Borzabadi S (2019). Clinical and metabolic response to probiotic administration in people with Parkinson’s disease: a randomized, double-blind, placebo-controlled trial. Clin Nutr.

[129] Krüger JF, Hillesheim E, Pereira ACSN, Camargo CQ, Rabito EI (2021). Probiotics for dementia: a systematic review and meta-analysis of randomized controlled trials. Nutr Rev.

[130] Wang K, Wang K, Wang J, Yu F, Ye C, Fu Y (2022). Protective effect of Clostridium butyricum on Escherichia coli-induced endometritis in mice via ameliorating endometrial barrier and inhibiting inflammatory response. Microbiol Spectr.

[131] Sun J, Xu J, Yang B, Chen K, Kong Y, Fang N (2019). Effect of Clostridium butyricum against microglia-mediated neuroinflammation in Alzheimer’s disease via regulating gut microbiota and metabolites butyrate. Mol Nutr Food Res.

[132] Sun J, Li H, Jin Y, Yu J, Mao S, Su K-P (2021). Probiotic Clostridium butyricum ameliorated motor deficits in a mouse model of Parkinson’s disease via gut microbiota-GLP-1 pathway. Brain Behav Immun.

[133] Shin J, Noh J-R, Choe D, Lee N, Song Y, Cho S (2021). Ageing and rejuvenation models reveal changes in key microbial communities associated with healthy ageing. Microbiome.

[134] Ou Z, Deng L, Lu Z, Wu F, Liu W, Huang D (2020). Protective effects of Akkermansia muciniphila on cognitive deficits and amyloid pathology in a mouse model of Alzheimer’s disease. Nutr Diabetes.

[135] He X, Yan C, Zhao S, Zhao Y, Huang R, Li Y (2022). The preventive effects of probiotic Akkermansia muciniphila on D-galactose/AlCl3 mediated Alzheimer’s disease-like rats. Exp Gerontol.

[136] Blacher E, Bashiardes S, Shapiro H, Rothschild D, Mor U, Dori-Bachash M (2019). Potential roles of gut microbiome and metabolites in modulating ALS in mice. Nature.

[137] Nishiwaki H, Ito M, Hamaguchi T, Maeda T, Kashihara K, Tsuboi Y (2022). Short chain fatty acids-producing and mucin-degrading intestinal bacteria predict the progression of early Parkinson’s disease. NPJ Parkinson’s Disease.

[138] Amorim Neto DP, Bosque BP, Pereira de Godoy JV, Rodrigues PV, Meneses DD, Tostes K (2022). Akkermansia muciniphila induces mitochondrial calcium overload and α -synuclein aggregation in an enteroendocrine cell line. iScience.

[139] Cuervo-Zanatta D, Syeda T, Sánchez-Valle V, Irene-Fierro M, Torres-Aguilar P, Torres-Ramos MA (2022). Dietary fiber modulates the release of gut bacterial products preventing cognitive decline in an Alzheimer’s mouse model. Cell Mol Neurobiol.

[140] Abdel-Haq R, Schlachetzki JCM, Boktor JC, Cantu-Jungles TM, Thron T, Zhang M (2022). A prebiotic diet modulates microglial states and motor deficits in α-synuclein overexpressing mice. eLife.

[141] Gubert C, Kong G, Costello C, Adams CD, Masson BA, Qin W (2024). Dietary fibre confers therapeutic effects in a preclinical model of Huntington’s disease. Brain Behav Immun.

[142] Gu Y, Nishikawa M, Brickman AM, Manly JJ, Schupf N, Mayeux RP (2021). Association of dietary prebiotic consumption with reduced risk of Alzheimer’s disease in a multiethnic population. Curr Alzheimer Res.

[143] Astarloa R, Mena MA, Sánchez V, de la Vega L, de Yébenes JG (1992). Clinical and pharmacokinetic effects of a diet rich in insoluble fiber on Parkinson disease. Clin Neuropharmacol.

[144] Jiang Y, Li K, Li X, Xu L, Yang Z (2021). Sodium butyrate ameliorates the impairment of synaptic plasticity by inhibiting the neuroinflammation in 5xFAD mice. Chem Biol Interact.

[145] Wang C, Zheng D, Weng F, Jin Y, He L (2021). Sodium butyrate ameliorates the cognitive impairment of Alzheimer’s disease by regulating the metabolism of astrocytes. Psychopharmacology.

[146] Liu J, Li H, Gong T, Chen W, Mao S, Kong Y (2020). Anti-neuroinflammatory effect of short-chain fatty acid acetate against Alzheimer’s disease via upregulating GPR41 and inhibiting ERK/JNK/NF-κB. J Agric Food Chem.

[147] Lang W, Li X, Wang Y, Duan Y, Wang Y, Wei P (2022). Sodium propionate improves cognitive and memory function in mouse models of Alzheimer’s disease. Neurosci Lett.

[148] Neuffer J, González-Domínguez R, Lefèvre-Arbogast S, Low DY, Driollet B, Helmer C (2022). Exploration of the gut–brain axis through metabolomics identifies serum propionic acid associated with higher cognitive decline in older persons. Nutrients.

[149] Qiao C-M, Sun M-F, Jia X-B, Li Y, Zhang B-P, Zhao L-P (2020). Sodium butyrate exacerbates Parkinson’s disease by aggravating neuroinflammation and colonic inflammation in MPTP-induced mice model. Neurochem Res.

[150] Zhang Y-g, Wu S, Yi J, Xia Y, Jin D, Zhou J (2017). Target intestinal microbiota to alleviate disease progression in amyotrophic lateral sclerosis. Clin Ther.

[151] MahmoudianDehkordi S, Arnold M, Nho K, Ahmad S, Jia W, Xie G (2018). Altered bile acid profile associates with cognitive impairment in Alzheimer’s disease—an emerging role for gut microbiome. Alzheimers Dement.

[152] Chen S-J, Chen C-C, Liao H-Y, Wu Y-W, Liou J-M, Wu M-S (2022). Alteration of gut microbial metabolites in the systemic circulation of patients with Parkinson’s disease. J Parkinsons Dis.

[153] Mulak A (2021). Bile acids as key modulators of the brain-gut-microbiota axis in Alzheimer’s disease. J Alzheimers Dis.

[154] Chen L, Chen Y, Zhao M, Zheng L, Fan D (2020). Changes in the concentrations of trimethylamine N-oxide (TMAO) and its precursors in patients with amyotrophic lateral sclerosis. Sci Rep.

[155] Quan W, Qiao C-M, Niu G-Y, Wu J, Zhao L-P, Cui C (2023). Trimethylamine N-oxide exacerbates neuroinflammation and motor dysfunction in an acute MPTP mice model of Parkinson’s disease. Brain Sci.

[156] Zhang L, Yu F, Xia J (2023). Trimethylamine N-oxide: role in cell senescence and age-related diseases. Eur J Nutr.

[157] Gao Q, Wang Y, Wang X, Fu S, Zhang X, Wang R-T (2019). Decreased levels of circulating trimethylamine N-oxide alleviate cognitive and pathological deterioration in transgenic mice: a potential therapeutic approach for Alzheimer’s disease. Aging.

[158] Li J, Zhang L, Wu T, Li Y, Zhou X, Ruan Z (2020). Indole-3-propionic acid improved the intestinal barrier by enhancing epithelial barrier and mucus barrier. J Agric Food Chem.

[159] Fang H, Fang M, Wang Y, Zhang H, Li J, Chen J (2022). Indole-3-propionic acid as a potential therapeutic agent for sepsis-induced gut microbiota disturbance. Microbiol Spectr.

[160] Bendheim PE, Poeggeler B, Neria E, Ziv V, Pappolla MA, Chain DG (2002). Development of indole-3-propionic acid (oxigon™) for Alzheimer’s disease. J Mol Neurosci.

[161] Sekikawa A, Wharton W, Butts B, Veliky CV, Garfein J, Li J (2022). Potential protective mechanisms of S-equol, a metabolite of soy isoflavone by the gut microbiome, on cognitive decline and dementia. Int J Mol Sci.

[162] Johnson SL, Park HY, Vattem DA, Grammas P, Ma H, Seeram NP (2020). Equol, a blood–brain barrier permeable gut microbial metabolite of dietary isoflavone daidzein, exhibits neuroprotective effects against neurotoxins induced toxicity in human neuroblastoma SH-SY5Y cells and Caenorhabditis elegans. Plant Foods Hum Nutr.

[163] Cecarini V, Cuccioloni M, Zheng Y, Bonfili L, Gong C, Angeletti M (2021). Flavan-3‐ol microbial metabolites modulate proteolysis in neuronal cells reducing amyloid‐beta (1‐42) levels. Mol Nutr Food Res.

[164] Sipilä PN, Heikkilä N, Lindbohm JV, Hakulinen C, Vahtera J, Elovainio M (2021). Hospital-treated infectious diseases and the risk of dementia: a large, multicohort, observational study with a replication cohort. Lancet Infect Dis.

[165] Bruno F, Malvaso A, Canterini S, Bruni AC (2022). Antimicrobial peptides (AMPs) in the pathogenesis of Alzheimer’s disease: implications for diagnosis and treatment. Antibiot (Basel).

[166] Minter MR, Hinterleitner R, Meisel M, Zhang C, Leone V, Zhang X (2017). Antibiotic-induced perturbations in microbial diversity during post-natal development alters amyloid pathology in an aged APP_SWE_/PS1_∆E9_ murine model of Alzheimer’s disease. Sci Rep.

[167] Mosaferi B, Jand Y, Salari A-A (2021). Antibiotic-induced gut microbiota depletion from early adolescence exacerbates spatial but not recognition memory impairment in adult male C57BL/6 mice with Alzheimer-like disease. Brain Res Bull.

[168] Goetghebeur PJD, Wesnes KA, Targum SD (2019). D-cycloserine improves difficult discriminations in a pattern separation task in Alzheimer’s disease patients with dementia. J Alzheimers Dis.

[169] Kim M, Park SJ, Choi S, Chang J, Kim SM, Jeong S (2022). Association between antibiotics and dementia risk: a retrospective cohort study. Front Pharmacol.

[170] Ternák G, Németh M, Rozanovic M, Bogár L (2022). Alzheimer’s disease-related dysbiosis might be triggered by certain classes of antibiotics with time-lapse: new insights into the pathogenesis?. J Alzheimers Dis.

[171] Molloy DW, Standish TI, Zhou Q, Guyatt G (2012). A multicenter, blinded, randomized, factorial controlled trial of doxycycline and rifampin for treatment of Alzheimer’s disease: the DARAD trial. Int J Geriatr Psychiatry.

[172] Cui C, Hong H, Shi Y, Zhou Y, Qiao C-M, Zhao W-J (2022). Vancomycin pretreatment on MPTP-induced Parkinson’s disease mice exerts neuroprotection by suppressing inflammation both in brain and gut. J Neuroimmune Pharmacol.

[173] Zhou X, Lu J, Wei K, Wei J, Tian P, Yue M et al. Neuroprotective effect of ceftriaxone on MPTP-induced Parkinson’s disease mouse model by regulating inflammation and intestinal microbiota. Oxid Med Cell Longev. 2021; 2021:1–15. 10.1155/2021/9424582 PMC8687851 34938384

[174] Hong C-T, Chan L, Chen K-Y, Lee H-H, Huang L-K, Yang Y-CSH (2022). Rifaximin modifies gut microbiota and attenuates inflammation in Parkinson’s disease: preclinical and clinical studies. Cells.

[175] Investigators NN-P (2006). A randomized, double-blind, futility clinical trial of creatine and minocycline in early Parkinson disease. Neurology.

[176] Mertsalmi TH, Pekkonen E, Scheperjans F (2019). Antibiotic exposure and risk of Parkinson’s disease in Finland: a nationwide case-control study. Mov Disord.

[177] Sun J, Zhan Y, Mariosa D, Larsson H, Almqvist C, Ingre C (2019). Antibiotics use and risk of amyotrophic lateral sclerosis in Sweden. Eur J Neurol.

[178] Twort FW (1915). An investigation on the nature of ultra-microscopic viruses. Lancet.

[179] Duan Y, Young R, Schnabl B (2021). Bacteriophages and their potential for treatment of gastrointestinal diseases. Nat Rev Gastroenterol Hepatol.

[180] Teng Y, Mu J, Xu F, Zhang X, Sriwastva MK, Liu QM (2022). Gut bacterial isoamylamine promotes age-related cognitive dysfunction by promoting microglial cell death. Cell Host Microbe.

[181] Zhang X, Luo X, Tian L, Yue P, Li M, Liu K (2023). The gut microbiome dysbiosis and regulation by fecal microbiota transplantation: Umbrella review. Front Microbiol.

[182] Sun J, Xu J, Ling Y, Wang F, Gong T, Yang C (2019). Fecal microbiota transplantation alleviated Alzheimer’s disease-like pathogenesis in APP/PS1 transgenic mice. Transl Psychiatry.

[183] Elangovan S, Borody TJ, Holsinger RMD (2022). Fecal microbiota transplantation reduces pathology and improves cognition in a mouse model of Alzheimer’s disease. Cells.

[184] Zhao Z, Ning J, Bao X-q, Shang M, Ma J, Li G (2021). Fecal microbiota transplantation protects rotenone-induced Parkinson’s disease mice via suppressing inflammation mediated by the lipopolysaccharide-TLR4 signaling pathway through the microbiota-gut-brain axis. Microbiome.

[185] Park S-H, Lee J-H, Kim J-S, Kim TJ, Shin J, Im JH (2022). Fecal microbiota transplantation can improve cognition in patients with cognitive decline and Clostridioides difficile infection. Aging.

[186] Cheng Y, Tan G, Zhu Q, Wang C, Ruan G, Ying S (2023). Efficacy of fecal microbiota transplantation in patients with Parkinson’s disease: clinical trial results from a randomized, placebo-controlled design. Gut Microbes.

[187] Xue L-J, Yang X-Z, Tong Q, Shen P, Ma S-J, Wu S-N (2020). Fecal microbiota transplantation therapy for Parkinson’s disease. Medicine (Baltimore).

[188] Sorbara MT, Pamer EG (2022). Microbiome-based therapeutics. Nat Rev Microbiol.

[189] Cheng AG, Ho P-Y, Aranda-Díaz A, Jain S, Yu FB, Meng X (2022). Design, construction, and in vivo augmentation of a complex gut microbiome. Cell.

[190] Fong W, Li Q, Yu J (2020). Gut microbiota modulation: a novel strategy for prevention and treatment of colorectal cancer. Oncogene.

[191] Hou M, Xu G, Ran M, Luo W, Wang H (2021). APOE-ε4 carrier status and gut microbiota dysbiosis in patients with Alzheimer disease. Front Neurosci.

[192] Cammann D, Lu Y, Cummings MJ, Zhang ML, Cue JM, Do J (2023). Genetic correlations between Alzheimer’s disease and gut microbiome genera. Sci Rep.

[193] Yan Y, Ren S, Duan Y, Lu C, Niu Y, Wang Z (2021). Gut microbiota and metabolites of α-synuclein transgenic monkey models with early stage of Parkinson’s disease. NPJ Biofilms Microbiomes.

[194] Fassarella M, Blaak EE, Penders J, Nauta A, Smidt H, Zoetendal EG (2021). Gut microbiome stability and resilience: elucidating the response to perturbations in order to modulate gut health. Gut.

